# Uncovering nonlinear causal relationship between public environmental concern and air pollution in China using convergent cross mapping

**DOI:** 10.1038/s41598-026-50678-w

**Published:** 2026-04-27

**Authors:** Qiao Wang, Keliang Long, Bo Wu

**Affiliations:** 1 School of Finance, Zhongnan University of Economics and Law, Wuhan, 430073 China; 2 School of Statistics and Mathematics, Zhongnan University of Economics and Law, Wuhan, 430073 China; 3 College of Mathematics and Statistics, Jishou University, Jishou, 416000 China

**Keywords:** public environmental concern, Convergent Cross Mapping, causal relationship, feedback mechanism, Environmental sciences, Environmental social sciences

## Abstract

Previous studies have investigated the relationship between public environmental concern (PEC) and air pollution using annual-scale data. Due to the nonlinear and abrupt nature of public perception and response to sudden events, traditional methods may obscure the true causal relationship between PEC and air pollution. In this paper, we extend the measurement of PEC and use the Convergent Cross Mapping (CCM) to analyze its causal relationship with air pollution. Based on daily average PEC, AQI and PM_2.5_ data from January 1, 2023 to December 31, 2024 for 115 prefecture-level cities in China, we find that the relationship between PEC and air pollution exhibits nonlinear and weak coupling characteristics. At the same time, cities showing a significant causal effect of PEC on air pollution are predominantly situated in the North China Plain and the Yangtze River Economic Belt, regions characterized by relatively high levels of economic development. Compared to PEC affecting air pollution, air pollution significantly influences PEC in more cities. This result implies a reversed causal relationship in most cities, whereby air pollution is not alleviated by increased public environmental concern, but instead stimulates greater public attention to environmental issues through its adverse health impacts. PEC is effective as a pollution mitigation strategy only in a limited number of economically advanced cities. It is recommended that relevant departments introduce feasible policies that facilitate public feedback and reinforce the enforcement of environmental regulations and penalties. Relevant agencies should develop a more effective response framework, manage public attention data in a systematic and scientific manner, and explore region-specific approaches to utilize public environmental awareness to mitigate air pollution.

**Supplementary Information:**

The online version contains supplementary material available at 10.1038/s41598-026-50678-w.

## Introduction

With the rapid advancement of the economy and education, public expectations for living environment quality have increased, leading to growing concern over environmental issues^1,2^. Public environmental concern (PEC), as a “bottom-up” feedback mechanism, plays a supervisory role in government pollution control efforts^3^. The revision of the Environmental Protection Law of the People’s Republic of China included “information disclosure and public participation” for the first time in 2014. In 2015, the Ministry of Environmental Protection issued the “Measures for Public Participation in Environmental Protection.” Since 2018, the Ministry of Ecology and Environment of China has held monthly press briefings to respond to PEC. Public concern for the environment has promoted revisions PM_2.5_ concentration standards and monitoring methods in China^4^. The Chinese government has sought to involve the public in formulating pollution control strategies to improve air quality and increase residents’ satisfaction with their living environment. If the logic that PEC leads to improvements in air quality holds, then PEC can be regarded as an effective feedback mechanism in mitigating air pollution. However, if there is no significant causal relationship between PEC to air pollution, then the feedback mechanism of PEC is ineffective. Therefore, accurately identifying the interactive effects between PEC and air pollution is essential for urban managers to reassess the causal chain of environmental governance.

The relationship between PEC and air pollution has received widespread attention in the academic community. In theory, they are often regarded as being interrelated and mutually causal. On the one hand, PEC serves as a bottom-up feedback channel that can reduce air pollution. Public attention to environmental issues places tremendous pressure on the government^3,5^. To respond to the public’s legitimate demands for air pollution reduction, the government will adopt stricter environmental regulations and penalties^6^. Stricter environmental laws are being introduced to regulate pollutants such as industrial sulfur dioxide, atmospheric emissions, and wastewater discharge^7^. At the same time, the government will also more rigorously enforce existing environmental laws to meet public demands for environmental protection^8^. Imposing fines is one of the main tools used to penalize companies for non-compliant emissions. By increasing the severity of penalties, the government can motivate firms to alter their behavior^9^. Under the combined effects of governmental pressure and incentives, enterprises will enhance production and operational efficiency and corporate responsibility through technological innovation, thereby achieving pollution and carbon reduction. On the other hand, worsening air pollution can intensify public concern about environmental issues. In daily life, noticeable physical effects of pollution, like blurred visibility and offensive smells, are key factors drawing public attention^10^. Moreover, severe air pollution can cause significant harm to human health, including lung cancer, heart disease, respiratory illnesses, and hypertension^7,11^. These conditions lead to symptoms such as shortness of breath, coughing, and asthma, which in turn increase public dissatisfaction with local pollution and raise awareness.

Existing studies have recognized the complementary monitoring role of social media data in environmental pollution, as well as its advantages over traditional interviews and surveys in terms of coverage and update frequency^6^. Using social media data such as Weibo and Baidu search as proxy indicators of PEC, some studies have employed single-equation linear regression models to examine their relationship with air pollution, finding that PEC can effectively reduce air pollution levels in the short term^12,13^. Nevertheless, the assumption of linear models in this line of research presents several limitations. First, unlike relatively stable stochastic systems in economics, air pollution results from the combined effects of meteorological conditions, topography, emissions, and other factors, leading to complex dynamic relationships among variables, and its time series exhibits significant nonlinearity and non-stationarity^2,14,15^. Second, the temporal resolution in existing studies is relatively coarse, as most analyses rely on annual data, overlooking the heterogeneous patterns of air pollution and public environmental concern across daily, monthly, and quarterly timescales. For example, public sensitivity to PM_2.5_ pollution is typically higher in winter; mild pollution may not stimulate online searches or postings, whereas severe pollution can cause sudden surges in search activity and social media engagement, leading to nonlinear responses to extreme events. Therefore, the linear model assumptions in existing studies may obscure the true causal relationship between the two.

Identifying causal relationships between variables is one of the most challenging goals in scientific research^16^. Traditional studies often use experimental approaches to examine causal relationships, but these methods are often impractical due to ethical, legal, and operational constraints^17^. At present, drawing causal inferences from observational data is a widely adopted and valuable method across diverse scientific domains^18–20^. Granger (1969) proposed a method for detecting causality in time series based on predictive power, which is widely regarded as the most prevalent causality testing tool^18^. Although the Granger causality test has been widely used across various disciplines, its inherent limitations have gradually become evident during practical applications^19^. The first limitation is linearity. Granger causality applies to linear stochastic systems, whereas most real-world systems are nonlinear^20^. Second is separability, which is a core assumption of the Granger causality test. In nonlinear systems, if X causes Y, information about X is embedded in Y and cannot be separated from the full set of potential causal variables of Y. In other words, nonlinear systems are non-separable, and existing studies have confirmed that PEC and air pollution mutually influence each other^3^. A third limitation is that Granger causality is suited for detecting strongly coupled relationships, but tends to perform poorly in identifying weak or subtle causal interactions. Fourth, when two variables are simultaneously influenced by a common factor, Granger causality struggles to distinguish between causation and correlation. The Granger test relies on the specification of econometric models, yet in reality, variables within systems are not simply connected, making direct modeling through standard equations inappropriate^20^. Model-driven methods have been criticized by scholars^21,22^. Specifically, for the research topic of this study, most existing works assume a linear relationship between PEC and air pollution for empirical analysis, which contradicts the nonlinear nature of the problem^4^. Some studies use cubic functions to characterize nonlinearity, but this also leads to model misspecification, as do simultaneous equation models^5^. To overcome the limitations of Granger causality, Sugihara et al. proposed a data-driven method called Convergent Cross Mapping (CCM), which offers a new way to uncover nonlinear causal relationships among weakly coupled variables^20^. CCM is a nonparametric method for detecting bidirectional causality from time series data; unlike traditional methods based on linear regression or Granger causality, it does not rely on the separability assumption and is particularly suitable for analyzing weakly coupled nonlinear dynamic systems. To address endogeneity caused by bidirectional causality in traditional econometric models, CCM does not prespecify causal directions or construct regression equations, but instead identifies causality through the intrinsic dynamics of the data, thereby fundamentally avoiding model-induced endogeneity issues. CCM has been successfully applied in various fields such as environmental studies, ecology, environmental science, biology, and geography^16,22^.

The daily average PEC, PM_2.5_ and AQI data are used in this study, and CCM is applied to identify the causal relationship between urban PEC and air pollution, aiming to assess whether environmental concern acts as a feedback mechanism to mitigate air pollution. From a methodological perspective, this study accounts for the nonlinear and weak coupling characteristics between PEC and air pollution, and is the first to apply CCM to this interaction, thus deepening the discourse on their interdependence. From a policy perspective, this study critically examines and reassesses the role of PEC as a feedback mechanism within the framework of environmental governance. It offers both theoretical insights and empirical evidence to support more nuanced and targeted policymaking in the context of urban air pollution control.

## Literature review

### Public environmental concern

Public environmental concern (PEC) refers to the level of public attention to social and environmental issues through various channels and platforms. The development of mobile internet and social media has provided efficient and accessible platforms for the public to obtain environmental information, express concerns, and participate in environmental protection actions. The transparency and visualization of air pollution information (e.g., AQI apps and real-time government platforms) have shifted environmental risk perception from indirect to direct. Meanwhile, the aggregation and diffusion of environmental events on social media platforms have significantly expanded both the scope and intensity of PEC, triggering emotional resonance and public opinion amplification. The public are not only recipients of environmental information, but also its monitors, and can contact relevant authorities and provide feedback through *VoIP*, Weibo, WeChat^23^. For example, during the winter of 2016, northern China experienced several days of smog, sparking heated debate and complaints on Weibo, with the public calling for stricter environmental regulations and a “war on pollution” campaign. Direct searches and disclosures of environmental issues by the public on internet platforms can be regarded as the formation of PEC. Previously, academic studies primarily measured public environmental concern using mainstream feedback indicators such as the number of National People’s Congress proposals, total letters and visits, environmental petition letters, and Chinese People’s Political Consultative Conference proposals. According to the Methodology for Public Participation in Environmental Impact Assessment, PEC also serves as a means for citizens to participate in social and environmental improvement^24^. Depending on regional institutions and customs, residents can engage in environmental concern either offline or online through communication or the internet^25^. Offline public participation in environmental concern includes forming related social organizations, direct communication and negotiation with polluters, and green consumption or investment behaviors. As public concern can be visualized using search engine statistics, current researchers represent public attention to environmental issues through various search indices^11,26^. As the largest Chinese-language search engine, Baidu has developed an extensive search index that reflects public concern on various topics via keyword tracking. Guo et al. (2020) used the Baidu Index to measure investor attention to environmental issues and assessed a series of environmental policies from 2014 to 2017 ^27^.

PEC has transitioned from a static emotional state to a quantifiable and modelable dynamic social variable. Exploring the dynamic coupling relationship and potential causal mechanisms between PEC and pollution levels is of great theoretical and practical significance for understanding the “social feedback loop” in environmental governance and building a modern governance system centered on public participation.

### The interaction between PEC and air pollution

The PEC can effectively improve air pollution^12,13,28^. Public concern about the environment places considerable pressure on the government^3^. To meet the public’s legitimate demands for pollution reduction, the government adopts stricter environmental regulations^6^, such as regulating the discharge of industrial waste gas and wastewater, which can effectively improve air quality^7,8^. The dual constraints of economic growth and environmental improvement have led to a divergence between environmental regulation and its enforcement. Public environmental concern plays a key role in monitoring and ensuring the enforcement of environmental regulations^3^. Li et al. (2022), using air pollution data and the Baidu Index, found that public concern can notably improve air quality by pressuring the government and voicing discontent over environmental and health issues^13^. Wu et al. (2021) studied the impact of public concern on haze reduction, revealing that while such concern reduces haze pollution, its effectiveness varies across regions^29^. Li et al. (2021) analyzed air pollution data from 13 Chinese cities between 2014 and 2019 to examine the impact of public concern on haze pollution^12^. The results indicate that environmental concern among the public has a notable short-term effect in reducing haze pollution.

Moreover, worsening air pollution may increase the level of public concern for environmental issues^27,30^. The physical and sensory changes caused by pollution are among the main drivers of public concern about environmental issues^10^. The worsening of air pollution is often accompanied by problems such as blurred vision and unpleasant odors, which sharply heighten public concern for the environment. In addition, air pollution may pose threats to public health, including heart disease, respiratory illnesses, and hypertension^7,11^. Faced with health risks from air pollution, residents tend to express strong environmental demands. For example, during the Xiamen PX project incident, the public responded to health threats by voicing resistance through public opinion as a form of feedback to the government^31^. The severe air pollution in 2013 across Beijing and nearby regions caused a spike in respiratory illnesses and heightened public awareness and anxiety regarding environmental problems^32,33^. With rising economic levels, public demand has shifted from basic survival needs to a focus on health and quality of life. This transition has raised expectations for clean air, a healthy ecological environment, and public safety, making severe air pollution a key driver of environmental concern. As public education improves, individuals become more capable of identifying and articulating environmental risks, which in turn intensifies collective reactions to deteriorating air quality.

Air pollution is affected by a wide array of factors, such as human emissions, climatic conditions, and terrain features. Public concern is not a primary driver of air pollution, and the reverse relationship also holds^34^. This implies that the association between public concern and air pollution levels is weak, with variations in pollution explaining only a small proportion of changes in public attention, indicating a lack of strong causality. Furthermore, the influence of public concern on air pollution depends on mechanisms such as governmental environmental regulation, and is subject to a lag in the “cognition–behavior” transformation, thereby exhibiting weak coupling characteristics at the daily scale. Finally, public attention to a given issue typically follows a “peak–decay” cyclical pattern. In the early stage of major pollution events, public attention may increase sharply; however, if pollution persists and governance outcomes remain limited, “attention fatigue” may occur, leading to a decline in search activity even when pollution levels remain high. This psychological adaptation mechanism further weakens the synchrony between pollution levels and public concern, resulting in weak coupling between PEC and air pollution.

## Materials and methods

### Air pollution

In this study, hourly AQI and PM_2.5_ data were collected from the China National Environmental Monitoring Center (http://www.cnemc.cn/). The selected city sample covers a wide range and effectively preserves the regional heterogeneity of Chinese cities, demonstrating good statistical representativeness. Furthermore, according to the Technical Regulation on Ambient Air Quality Index, we calculated the daily average AQI and PM_2.5_ for each city from January 1, 2023 to December 31, 2024. To ensure data quality, prefecture-level cities with severe missing data were excluded, resulting in a final sample of 115 cities.

### Public environmental concern

Public environmental concern (PEC) is a core concept in environmental governance, and its theoretical connotation has been a key focus of academic research. Currently, there are two main perspectives on the definition of PEC. One perspective considers PEC equivalent to public environmental attitudes and concern, essentially reflecting subjective cognition and psychological tendencies toward environmental issues^35^. The other perspective emphasizes its action-oriented nature, arguing that PEC includes attitudinal concern, informal opinion expression, and formal environmental participation, and may extend to concrete pro-environmental actions^36^. It is widely acknowledged that PEC has a layered structure, reflecting a gradient transition from fundamental psychological concern to advanced forms of behavioral engagement. The degree of public concern for the environment constitutes the prerequisite for the formation of PEC. Institutionalized participation and organized civic actions are the outcomes of PEC transformation. Fundamentally, PEC embodies public risk perception, attention distribution, and informal attitude expression toward environmental issues, acting as a linkage between environmental cognition and governance practices. Currently, there are three main types of proxy variables for PEC. Survey-based data can directly reflect public environmental attitudes but suffer from limitations in sample size, temporal continuity, and regional comparability. Data on formal environmental participation primarily represent the advanced outcomes of PEC, making it difficult to capture its overall status and dynamic evolution. Big data sources such as the Baidu Search Index, with advantages of large samples, high frequency, and spatiotemporal comparability, have become mainstream measurement approaches. In response to concerns about the scientific validity of the Baidu Index, this study argues that environmental keyword search behavior directly reflects public risk perception and attention allocation. This aligns with the concept of PEC as a “bottom-up governance feedback mechanism.” It overcomes the limitations of survey data while avoiding the narrow interpretation inherent in formal participation data. In summary, this study defines PEC as public risk perception, attention allocation, and attitudinal expression toward air pollution. It is conceptualized as a bottom-up governance feedback mechanism based on information attention and online public discourse. This distinguishes it from institutionalized participation and organized actions. Using the Baidu Search Index as its proxy variable is consistent with the theoretical framework and compensates for the shortcomings of traditional indicators, ensuring both scientific validity and applicability.

The search index captures the active aspect of public concern, representing individuals who voluntarily spend time searching for environmental information online. According to the Research Report on Chinese Internet Users Search Behavior of Baidu in 2013 has a penetration rate of 96.3%, ranking first in China’s search engine market. Therefore, the Baidu Search Index serves as an effective proxy for measuring public attention to specific topics. Existing studies have used discussion data from social media platforms such as Weibo and WeChat as proxies for PEC; however, discussions on environmental topics on these platforms often exhibit an “event-driven” pattern, where major pollution incidents or the introduction of environmental policies lead to sharp surges in discussion intensity. During routine periods, however, discussion levels are relatively low, leading to datasets with frequent “zero values” or discontinuities, reflecting strong intermittent characteristics. To address this issue, later research has utilized the Baidu search frequency for PM_2.5_ as a surrogate indicator of environmental attention^26^. Other studies have identified the most common Chinese environmental terms from media reports and used their Baidu Search Index to measure PEC. However, these keywords are overly technical and mainly reflect the concern of more highly educated segments of the public. To more comprehensively reflect public concern about air pollution, we build on the above studies and use Baidu Search Indices for the keywords “PM_2.5_”, “air pollution”, “smog”, “haze”, “AQI”, and “air quality” to measure environmental concern. Specifically, we collected Baidu search index values for each keyword from January 11, 2023, to December 31, 2024, and calculated city-level averages to measure public concern about environmental issues. For the public, daily data have a stronger immediate impact than annual, monthly, or weekly data, as perceptual cognition is influenced by daily fluctuations, prompting timely responses. Moreover, data at larger temporal scales tend to smooth fluctuations, thereby obscuring the causal relationship between air pollution levels and public environmental concern. Therefore, this study employs daily data instead of annual data to accurately capture the short-term dynamic relationship between PM_2.5_ concentrations and public cognitive responses.

### Convergent cross mapping

The Convergent Cross Mapping (CCM) algorithm is a method for measuring causality between time series, developed based on state space reconstruction theory and Takens’ embedding theorem^37,38^. It measures the causality between time series by using predictions made through the reconstruction of phase space trajectories. The central principle of CCM is that if the state of the causal variable can be reconstructed from the time series it influences, then a causal link exists between them. Tsonis et al. (2015) noted that essential information about a multidimensional dynamical system can be obtained from any single time series within the system^39^. Based on Takens’ theorem, CCM can effectively determine whether two time series originate from the same dynamical system. If time series Y is causally influenced by X, the causal state of X can be reconstructed from Y, thereby revealing the causal relationship between X and Y. The degree of causality is quantified by the correlation between the reconstructed X values and the actual X time series. The more accurately X predicts values of Y, the stronger the causal influence from X to Y. If the prediction accuracy of the CCM algorithm increases with the length of the data, it indicates that X has a causal effect on Y.

The CCM algorithm primarily relies on Takens embedding theorem and the Simplex Projection method. The Simplex Projection approach is a proximity-based method that estimates kernel density in the reconstructed phase space by applying exponential weighting to the distances of neighboring points. For two equal-length sequences $\{ x(1),x(2), \cdots ,x(L)\}$ and $\{ y(1),y(2), \cdots ,y(L)\}$, each of length L. In the phase space reconstruction process, assuming E is the embedding dimension and τ is the sampling interval, the coordinates at time *t* are ${M_X}$ and ${M_Y}$, defined by the following formulas:

1 $$x(t)=\left\langle {X(t),X(t - \tau ),X(t - 2\tau ), \cdots ,X(t - (E - 1)\tau )} \right\rangle$$

2 $$y(t)=\left\langle {Y(t),Y(t - \tau ),Y(t - 2\tau ), \cdots ,Y(t - (E - 1)\tau )} \right\rangle$$

The reconstructed phase spaces $${M_X}$$ and $${M_Y}$$ can be derived from the coordinates $${M_X}=\{ \underset{\raise0.3em\hbox{$\smash{\scriptscriptstyle-}$}}{x} (t)\}$$ and $${M_Y}=\{ \underset{\raise0.3em\hbox{$\smash{\scriptscriptstyle-}$}}{y} (t)\}$$. $$\hat {Y}(t)|{M_X}$$is defined as the prediction obtained via CCM.

First, identify the vector$$x(t)$$ at time t on the .$${M_X}$$. coordinate and find its E + 1 nearest neighbors, naming them $$\underset{\raise0.3em\hbox{$\smash{\scriptscriptstyle-}$}}{x} ({t_1}) \cdots \underset{\raise0.3em\hbox{$\smash{\scriptscriptstyle-}$}}{x} ({t_{E+1}})$$ in order of increasing distance. Then, locate the mapped point $$\underset{\raise0.3em\hbox{$\smash{\scriptscriptstyle-}$}}{y} ({t_1}),\underset{\raise0.3em\hbox{$\smash{\scriptscriptstyle-}$}}{y} ({t_2}), \cdots ,\underset{\raise0.3em\hbox{$\smash{\scriptscriptstyle-}$}}{y} ({t_{E+1}})$$ corresponding to $$\underset{\raise0.3em\hbox{$\smash{\scriptscriptstyle-}$}}{x} ({t_1}) \cdots \underset{\raise0.3em\hbox{$\smash{\scriptscriptstyle-}$}}{x} ({t_{E+1}})$$ on the $${M_Y}$$ coordinate. Therefore, $$\hat {Y}(t)|{M_X}$$ can be obtained, as expressed by the following formula:

3 $$\hat {Y}(t)|{M_X}=\sum {{w_i}\underset{\raise0.3em\hbox{$\smash{\scriptscriptstyle-}$}}{y} ({t_i})} {\text{ }}i=1,2, \cdots ,E+1$$

where ${w_i}$ denotes the distance weight between $X(t)$and the i-th neighbor on the ${M_X}$ coordinate.

4 $${w_i}={u_i}/\sum {{u_j}{\text{ }}j=1,2, \cdots ,E+1}$$

5 $${u_i}=\exp \{ - d[\underset{\raise0.3em\hbox{$\smash{\scriptscriptstyle-}$}}{X} (t),\underset{\raise0.3em\hbox{$\smash{\scriptscriptstyle-}$}}{X} ({t_i})]/d[\underset{\raise0.3em\hbox{$\smash{\scriptscriptstyle-}$}}{X} (t),\underset{\raise0.3em\hbox{$\smash{\scriptscriptstyle-}$}}{X} ({t_1})]\}$$

where$$d[\underset{\raise0.3em\hbox{$\smash{\scriptscriptstyle-}$}}{X} (t),\underset{\raise0.3em\hbox{$\smash{\scriptscriptstyle-}$}}{X} ({t_1})]$$ denotes the Euclidean distance between $$\underset{\raise0.3em\hbox{$\smash{\scriptscriptstyle-}$}}{X} (t)$$ and $$\underset{\raise0.3em\hbox{$\smash{\scriptscriptstyle-}$}}{X} ({t_1})$$.

Furthermore, the strength of the causal relationship is quantified by calculating the correlation coefficient $\rho$ between $\hat {Y}(t)|{M_X}$ and $Y(t)$​. The formula is as follows:

6 $$\rho =\frac{{\sum\limits_{{i=1}}^{L} {(Y(i) - \bar {Y})(\hat {Y}(i)|{M_X} - \bar {\hat {Y}}\left| {{M_X}} \right.)} }}{{\sqrt {\sum\limits_{{i=1}}^{L} {{{(Y(i) - \bar {Y})}^2}{{(\hat {Y}(i)|{M_X} - \bar {\hat {Y}}\left| {{M_X}} \right.)}^2}} } }}$$

If sequence Y influences sequence X, the attractor manifold becomes denser as data length increases, reducing the distance to nearby points and making them closer to the true neighbors. As a result, $\{ \hat {Y}(t)|{M_X}\}$converges to $Y(t)$​, and the correlation coefficient $\rho$will lie in the range [0, 1].

In the CCM algorithm, $\rho$represents the strength of the causal relationship between sequence Y and sequence X. When$\rho =0$, it indicates that there is no causal relationship between Y and X. A larger value of $\rho$ implies a stronger causal relationship between sequence Y and sequence X.

## Results and discussion

### The nonlinear characteristics of PEC and air pollution

**Fig. 1:**
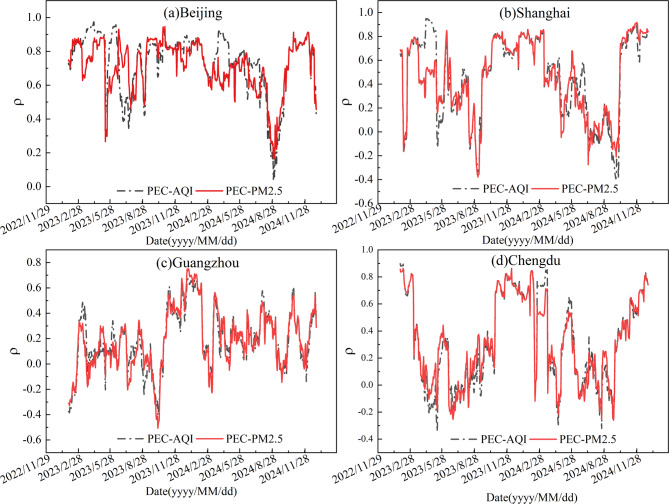
Nonlinear characteristics of PEC and air pollution.

**Fig. 2:**
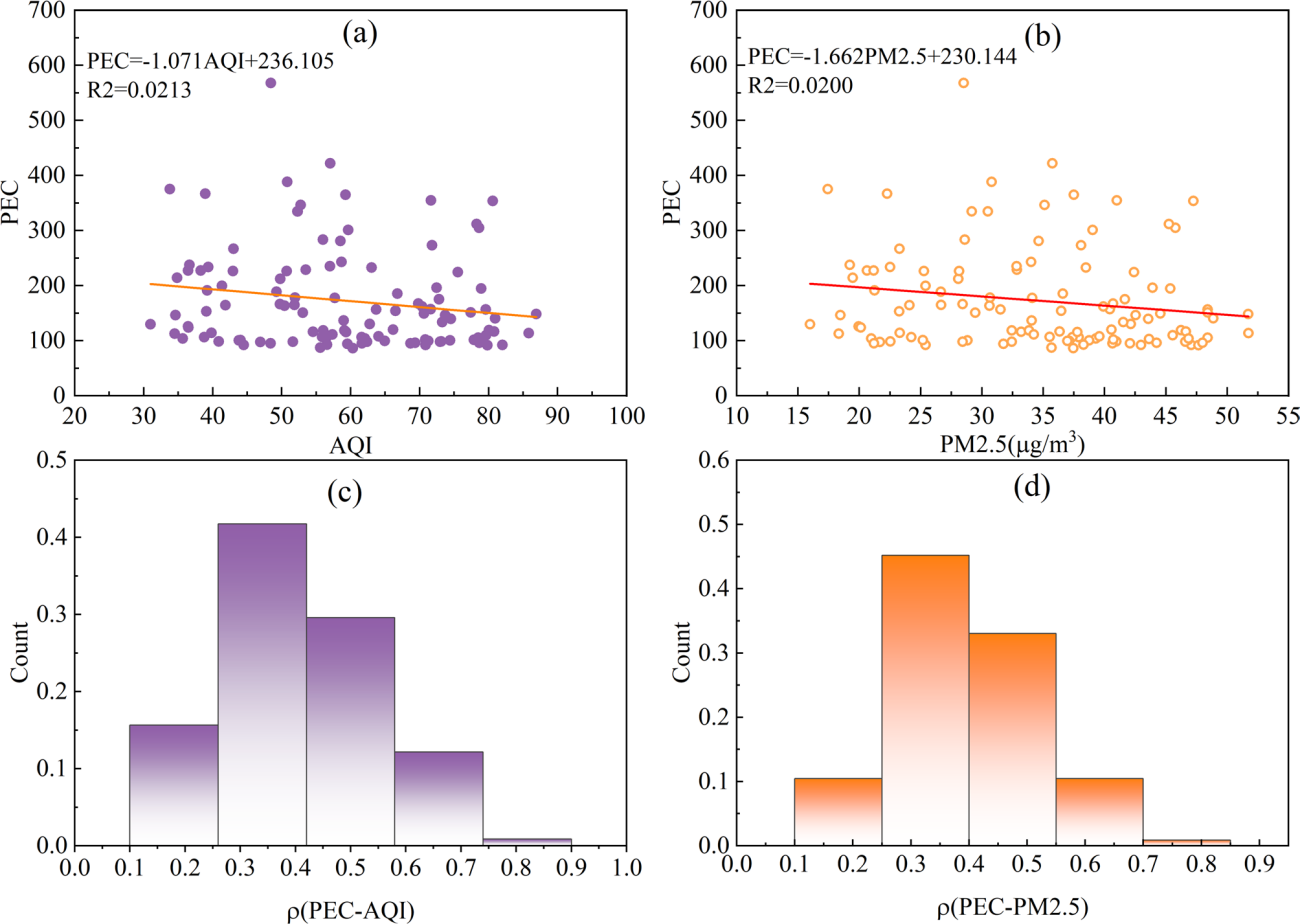
Weak coupled relationship between PEC and air pollution.

To investigate the nonlinear characteristics of Public Environmental Concerns (PEC), PM_2.5_, and AQI, a sliding Pearson correlation analysis was conducted, with the results shown in Fig. 1. Figure 1 illustrates the time-varying correlation curves between PEC and both AQI and PM_2.5_. The correlation between PEC and AQI (PM_2.5_) exhibits dynamic instability, alternating between presence and absence, and between positive and negative, which is a hallmark of nonlinear systems^40^. This may be primarily attributed to the inherent nonlinearity of air pollution, forming a weakly coupled nonlinear system with PEC. Furthermore, we explored the weak coupling characteristics of the dynamic system formed by PEC and AQI (PM_2.5_) across 115 prefecture-level cities in China, as illustrated in Fig. 2. As shown in Fig. 2(a) and 2(b), there is no significant correlation between PEC and AQI or PM_2.5_ pollution. Additionally, we calculated the correlation coefficients between PEC and AQI(PM_2.5_) for each of the 115 cities and visualized the distribution of these coefficients in Fig. 2(c) and 2(d). The results reveal that more than 60% of cities exhibit correlation coefficients between 0 and 0.3, signifying an evident weak coupling relationship. In summary, PEC and air pollution form a weakly coupled nonlinear system.

### Nonlinear response between PEC and air pollution

**Fig. 3:**
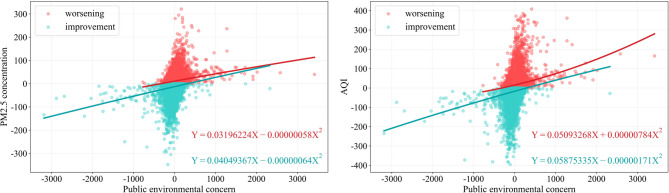
Response mechanism of PEC to changes in PM_2.5_ and AQI.

Figure 3 illustrates the nonlinear regression outcomes of PEC in response to variations in PM_2.5_ and AQI. As shown in Fig. 3, PEC exhibits a significant asymmetric response mechanism to changes in PM_2.5_ concentration and AQI, which can be characterized by the combined effects of the linear and quadratic terms. During the pollution deterioration stage, the linear coefficients of PEC are significantly positive, indicating that public concern increases markedly with worsening pollution. Specifically, the quadratic coefficient in the PM_2.5_ group is close to zero, exhibiting an approximately linear growth pattern; whereas in the AQI group, the quadratic coefficient is positive but small, indicating that public concern shows a rapid, stable, and sustained increase as pollution continues to worsen. In contrast, during the pollution improvement stage, the linear coefficients of PEC remain significantly positive and relatively larger, while the quadratic coefficients are negative, indicating a clear diminishing marginal effect. This result indicates that as air quality gradually improves, public environmental concern continues to increase but at a decreasing rate, exhibiting a transition from strong to weak growth dynamics. In summary, public responses to air pollution are markedly asymmetric across worsening and improving phases, where the former is characterized by rapid amplification of sensitivity, while the latter shows a gradual decline and attenuated responsiveness.

### Embedding dimension selection

The characteristics of the data are a prerequisite for method selection. The nonlinear and weakly coupled nature of PEC and AQI (PM_2.5_) renders traditional causality inference methods, such as the Granger causality test, unsuitable. Therefore, this study employs the CCM method to identify the causal relationship between PEC and air pollution. Prior to conducting the causality test, the optimal embedding dimension (E), the optimal lag (τ), and the nonlinear characteristics of the variables must be determined.

**Fig. 4:**
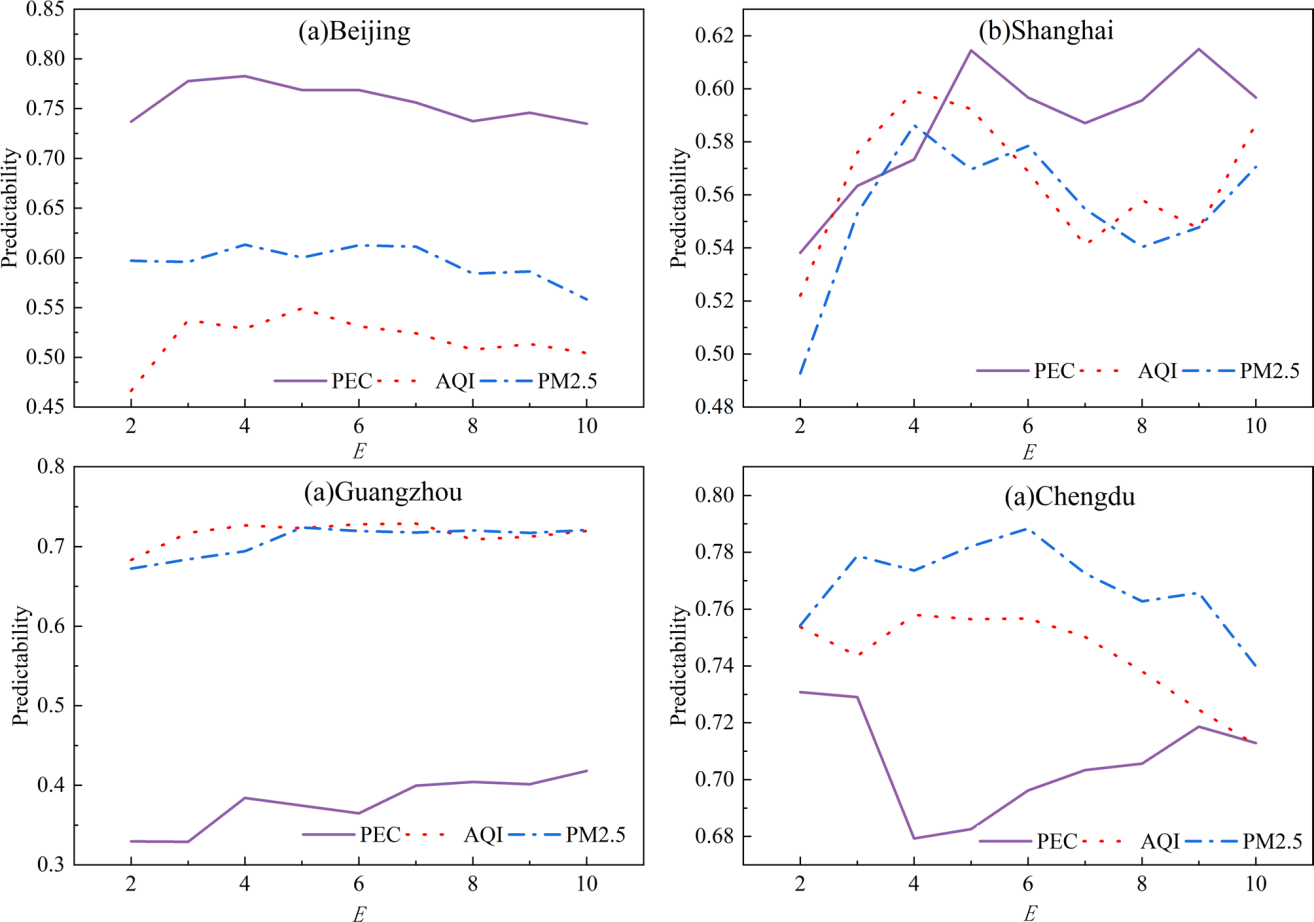
Optimal embedding dimension selection for PEC and air pollution.

First, the optimal embedding dimension (E) and the time lag (τ) for CCM testing are selected. The selection of the optimal embedding dimension is determined by the forecasting performance of each variable across various embedding levels. Predictive ability is measured by the correlation coefficient between observed and predicted values, which reaches its maximum at the optimal embedding dimension^40^. Accordingly, this study uses simplex projection to determine the optimal embedding dimensions for each variable in the selected sample cities. Figure 4 demonstrates the complete selection process for optimal embedding dimensions of PEC, AQI, and PM_2.5_ using Beijing, Shanghai, Guangzhou, and Chengdu as examples. The predictive performance for PEC, AQI, and PM_2.5_ in Beijing peaked at embedding dimensions of 4, 4, and 5, respectively. This indicates that the optimal embedding dimensions for PEC, AQI, and PM_2.5_ in Beijing are 4, 4, and 5, respectively. The optimal embedding dimensions for PEC, AQI, and PM_2.5_ in Shanghai are 5, 4, and 4, respectively. The optimal embedding dimensions for PEC, AQI, and PM_2.5_ in Guangzhou are 10, 4, and 5, respectively. The optimal embedding dimensions for PEC, AQI, and PM_2.5_ in Chengdu are 1, 4, and 6, respectively. Next, the time lag (τ) is determined. According to information theory, a larger lag results in greater information loss when reconstructing the shadow manifold, whereas a smaller lag enables higher-resolution cross mapping and more accurate testing results^41^. Therefore, to ensure more accurate testing, the lag τ is set to 1.

**Fig. 5:**
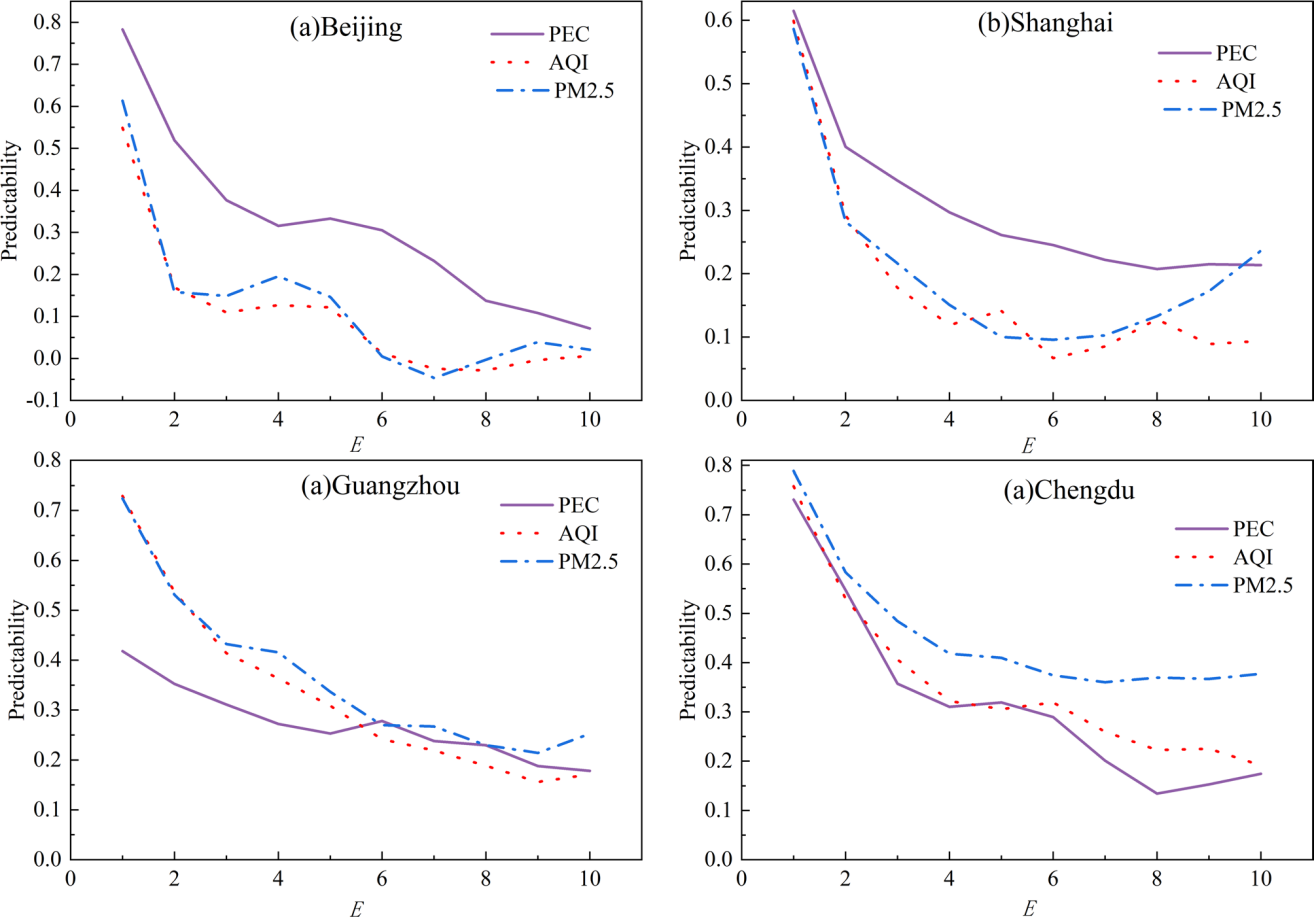
Testing the nonlinear characteristics of PEC and air pollution.

Secondly, the nonlinear characteristics of PEC, AQI and PM_2.5_ are tested. Despite ample empirical and theoretical evidence of air pollution’s nonlinearity, performing individual nonlinear tests for each variable within the CCM framework is still necessary. variables in nonlinear systems exhibit irregular fluctuations over time, and if a variable is nonlinear, predictions derived from short time lags are more accurate than those from longer lags^16^. In other words, the correlation between the predicted and actual values of the variable declines as the time lag increases. In this study, we apply the simplex projection method to test the nonlinear dynamics of PEC, AQI, and six specific air pollutants; the results are presented in Fig. 5. Figure 5 shows the trend in prediction skill for PEC, AQI, and PM_2.5_ across increasing time lags for Beijing, Shanghai, Guangzhou, and Chengdu. As shown in Fig. 5, the correlation between predicted and actual values of PEC, AQI, and PM_2.5_ decreases with increasing time intervals in all four cities. These results indicate that both PEC and AQI (PM_2.5_) exhibit clear nonlinear characteristics.

### The nonlinear causality between PEC and air pollution

**Fig. 6:**
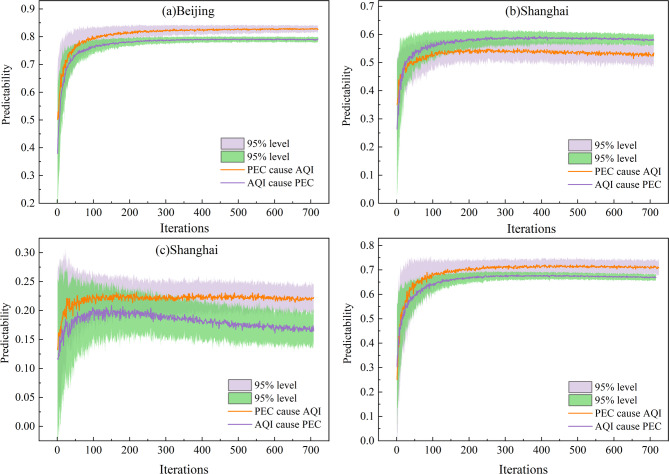
Results of causality test between PEC and AQI.

**Fig. 7:**
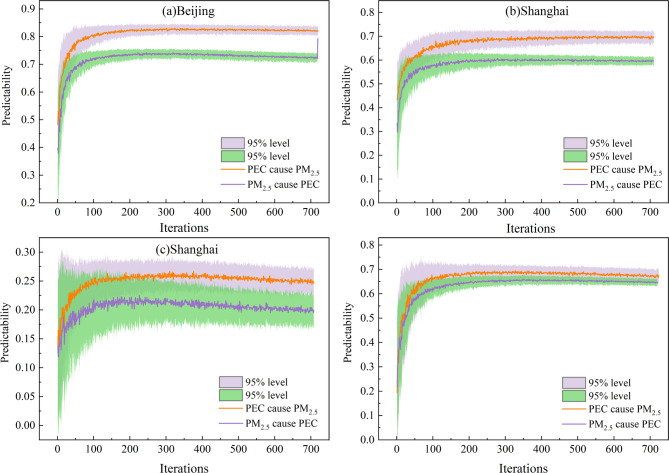
Results of causality test between PEC and PM_2.5_.

To identify causality between PEC and AQI (PM_2.5_) using the CCM method, it is necessary to examine whether the predictive skill of cross-mapping converges to a peak value. Since it is not feasible to present all CCM results, we selected four major cities (Beijing, Shanghai, Guangzhou, and Chengdu) based on PEC data from January 2023 to December 2024, and present their CCM causality test results in Figs. 6 and 7. As shown in Fig. 6, for Beijing, Shanghai, and Chengdu, the CCM correlation coefficients are generally high for both the causal direction from PEC to AQI and vice versa. Additionally, as the time series length increases, several CCM curves in these cities exhibit convergence to a single peak value. This strongly indicates that PEC and air pollution have a high mutual predictive capacity. For Guangzhou, the CCM correlation coefficients are generally low in both directions between PEC and AQI. With increased data length, some CCM curves in this city show a declining trend. This indicates that in Guangzhou, there is limited mutual predictive power between environmental concern and air pollution.

As shown in Fig. 7, the results of the CCM causality test between PEC and PM_2.5_ pollution are displayed. The results regarding the relationship between PEC and PM_2.5_ pollution are broadly similar to those with AQI. In Beijing, Shanghai, and Chengdu, the CCM correlation coefficients between PEC and PM_2.5_ are generally high. In Guangzhou, the CCM correlation values are consistently low. This result indicates that no causal relationship exists between PEC and PM_2.5_ in Guangzhou, while such a relationship is present in Beijing, Shanghai, and Chengdu. The Pearl River Delta has recently become a national frontrunner in air pollution mitigation efforts. Through the implementation of stricter pollution source controls, the PM_2.5_ concentration in the Pearl River Delta met national standards by 2015 and reached the WHO Interim Target 2 (WHO-II) level by 2020. In particular, Guangzhou has seen a significant reduction in PM_2.5_ concentrations^42^. In 2023, Guangzhou reported an average annual PM_2.5_ concentration of 23 µg/m³, down 56.6% from 2013, consistently meeting national standards for seven years and maintaining the best performance among core Chinese cities. Severe air pollution tends to trigger widespread attention by increasing physical perception and environmental awareness. Therefore, compared to Beijing, Shanghai, and Chengdu, Guangzhou’s good air quality may explain the absence of causality between PEC and AQI (PM_2.5_).

**Fig. 8:**
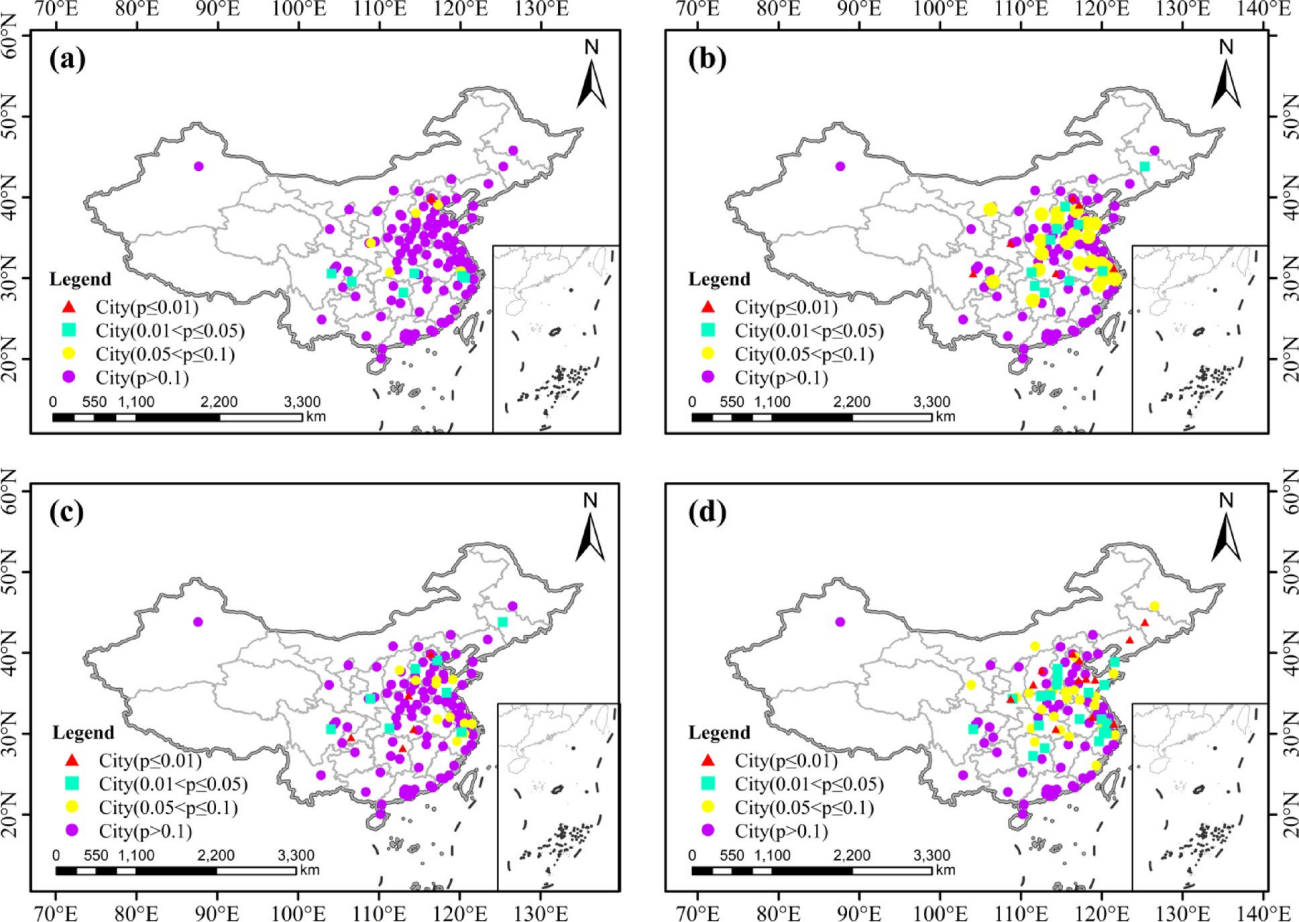
Significance levels of causality test coefficients between air pollution and PEC.

To further determine whether a causal relationship exists between PEC and air pollution, the statistical significance tests on the CCM correlation coefficients are used^16^. Figure 8 report the significance test results for PEC and AQI(PM_2.5_) across 115 cities.

As shown in Fig. 8(a), the CCM correlation between PEC and PM_2.5_ is statistically significant in 12 out of 115 cities at the 10% level, specifically in Beijing, Chengdu, Hangzhou, Huzhou, Shaoxing, Shijiazhuang, Tianjin, Wuhan, Xi’an, Yichang, Changsha, and Chongqing. At the 5% significance level, a significant effect of PEC on PM_2.5_ was observed in only seven cities: Beijing, Chengdu, Hangzhou, Shaoxing, Wuhan, Changsha, and Chongqing. At the 1% significance level, only Beijing exhibited a statistically significant influence of PEC on PM_2.5_. PEC can enhance the implementation of corporate pollution control strategies through citizen protests. This type of PEC can effectively increase the social visibility of corporate pollution, reduce firms’ bargaining power with local environmental authorities, and enable regulators to impose stronger penalties. Since stricter penalties can prompt behavioral change in firms, PEC can effectively reduce pollution levels^9^. Figure 8(b) presents the significance results of CCM coefficients for the effect of PM_2.5_ on PEC across 115 cities. As shown in Fig. 8(b), cities with significant CCM coefficients between PM_2.5_ and PEC are mainly located in the developed regions of the North China Plain and the Yangtze River Economic Belt. At the 10% significance level, PM_2.5_ was found to significantly influence PEC in 41 cities of China. At the 5% level, PM_2.5_ significantly affected PEC in 15 cities, namely Anyang, Baoding, Beijing, Changde, Chengdu, Huzhou, Jinan, Jiujiang, Shanghai, Tianjin, Wuhan, Xianyang, Yichang, Changchun and Changsha. At the 1% level, only six cities showed significant causal influence of PM_2.5_ on PEC, namely Beijing, Chengdu, Shanghai, Tianjin, Wuhan and Xianyang. In summary, at the 10%, 5%, and 1% significance levels, PM_2.5_ has a greater causal effect on PEC than vice versa in more cities. In daily life, perceptible physical changes caused by pollution (such as hazy vision and unpleasant odors) are primary triggers of public attention^10^. Therefore, individuals pay greater attention to air quality. Moreover, severe air pollution poses serious health risks, such as an increased likelihood of lung cancer, thereby heightening public concern and awareness of pollution^43,44^. Air pollution has been shown to elevate the risk of inflammation and oxidative stress^43,44^, which in turn contributes to a range of adverse respiratory outcomes, including dyspnea, persistent coughing, asthma, and chronic obstructive pulmonary disease. These health effects often necessitate increased use of medications and more frequent medical consultations, thereby enhancing public awareness and concern regarding air quality issues^44–47^. The emergence of these health risks and the public’s growing awareness of them will generate a ripple effect in society, leading to heightened PEC Conversely, increased environmental awareness among the public empowers citizens to exert pressure on the government, voice their dissatisfaction, and ultimately contribute to mitigating pollution and health-related threats^12^.

According to Fig. 8(c), with respect to effect of PEC on AQI, 24 out of 115 sample cities exhibit statistically significant CCM correlation coefficients at the 10% level, and these cities are mainly concentrated in the North China Plain and Yangtze River Economic Belt. At the 5% level, only 13 cities (including Beijing, Chengdu, Hangzhou, Linyi, Shijiazhuang, Tianjin, Wuhan, Xi’an, Yichang, Changchun, Changsha, Zhengzhou, and Chongqing) show a significant influence of PEC on AQI. At the stricter 1% level, only five cities (Beijing, Wuhan, Changsha, Zhengzhou, and Chongqing) exhibit a significant PEC-to-AQI causal relationship. Figure 8(d) reports the significance test results of CCM coefficients for effect of AQI on PEC across 115 cities. As indicated in Fig. 8(d), 57, 32, and 13 cities show statistically significant CCM coefficients for effect of AQI on PEC at the 10%, 5%, and 1% significance levels, respectively. In summary, the causal relationship between PEC and AQI is broadly consistent with that of PEC and PM_2.5_ in more cities. Compared to the influence of PEC on AQI, AQI affects PEC in a greater number of cities. Moreover, the cities where a significant causality exists between PEC and pollution are primarily situated in the economically developed areas of the North China Plain and the Yangtze River Economic Belt. This finding is consistent with previous research^30^, which suggests that residents in high-income and highly polluted cities tend to show the greatest concern for environmental issues.

Based on the above findings, this study further analyzes regional differences among cities in conjunction with existing literature. Existing studies indicate that higher socioeconomic status enhances environmental concern^48^. Individuals with higher income and better education have already satisfied basic material needs and therefore pay more attention to environmental quality^49^. According to post-materialist theory, well-educated and high-income individuals are more likely to embrace values that prioritize quality of life and environmental sustainability over economic growth and material accumulation^50^. From a regional development perspective, the causal effect of PEC on air pollution is mainly concentrated in economically developed cities in the North China Plain and the Yangtze River Economic Belt, essentially forming a “high attention-strong response” feedback loop once these regions reach a certain level in development stage, industrial structure, and urbanization. The North China Plain, as a traditional hub for heavy industry and energy production, has long depended on an extensive growth model characterized by high input and high emissions, thereby exacerbating the tension between environmental pollution and public health. Under this context, with the implementation of national strategies such as the coordinated development of the Beijing-Tianjin-Hebei region and joint air pollution prevention and control, cities in the North China Plain have accelerated their transition toward high-quality development, enabling governments to respond more promptly and effectively to public environmental demands, thereby transforming public concern into a driving force for pollution control. The Yangtze River Economic Belt is characterized by high urbanization, integrated urban agglomerations, and an open industrial system, exhibiting advantages such as high economic agglomeration and relatively optimized industrial structure; with relatively mature regional governance mechanisms, cities in this region have shifted their development goals from expanding traditional industries to achieving ecological livability and green sustainable growth, requiring governance capacity to match high public expectations, thereby making public environmental concern a sustained driver of emission reduction. At present, both regions have surpassed the basic survival-oriented development stage, with relatively high income and education levels, concentrated policy resources, and well-developed governance tools, leading to a significant increase in public environmental demands^51^. These factors collectively constitute the practical foundation through which PEC can influence air pollution. In contrast, other regions may face relatively lagging development stages, insufficient industrial support, and limited governance resources, leading the public to prioritize material development over environmental concerns^52^. Therefore, the causal effect of PEC on air pollution is primarily concentrated in economically developed cities in the North China Plain and the Yangtze River Economic Belt.

### Heterogeneity analysis

According to the Ambient Air Quality Standards (GB3095-2026), the primary daily average concentration limit of PM_2.5_, set at 25 µg/m³, is adopted as the threshold for classification. Furthermore, according to the Technical Regulation on Ambient Air Quality Index (HJ633-2026), an AQI value of 50 is used as the threshold. Subsequently, the daily average values of PM_2.5_ concentration and AQI for each city are calculated and grouped according to the standards. Under low pollution conditions, only 4 cities (AQI to PEC) and 1 city (PM_2.5_ to PEC) exhibit significant relationships. However, once the threshold is exceeded, the numbers increase to 53 cities for AQI to PEC and 40 cities for PM_2.5_ to PEC. The results indicate a clear threshold effect in public environmental concern responses to pollution changes, with limited variation at low pollution levels and significantly increased responsiveness once the threshold is exceeded. Based on the Ambient Air Quality Standard (GB3095-2026), the secondary daily mean PM_2.5_ threshold of 50 µg/m³ is adopted as the classification benchmark. If a city has more than 30% of days exceeding the secondary PM_2.5_ threshold within 730 days, it is considered to be in a long-term high-pollution environment and classified into the high-pollution group. According to this classification, 18 cities fall into the long-term high-pollution group. Among these 18 cities, only 2 exhibit a significant PEC to PM_2.5_ relationship. Additionally, according to the AQI technical regulation (HJ633-2026), AQI values between 201 and 300 indicate heavy pollution and values above 300 indicate severe pollution; thus, based on the target of controlling the proportion of such days within 1%, cities with more than 1% of days in heavy or severe pollution over 730 days are classified into the long-term high-pollution group, totaling 109 cities. Among these 109 cities, only 24 show a significant PEC to PM_2.5_ relationship. This indicates that in persistently polluted environments, public concern may diminish due to habituation or fatigue, thereby weakening its feedback effect.

### Robustness test

**Fig. 9:**
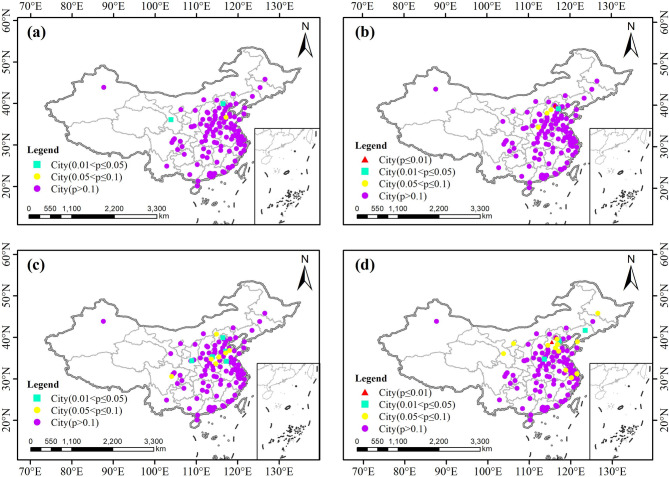
Significance levels of the causality test coefficients between weekly average air pollution and PEC: (**a**) PEC to PM_2.5_; (**b**) PM_2.5_ to PEC; (**c**) PEC to AQI; (**d**) AQI to PEC.

**Fig. 10:**
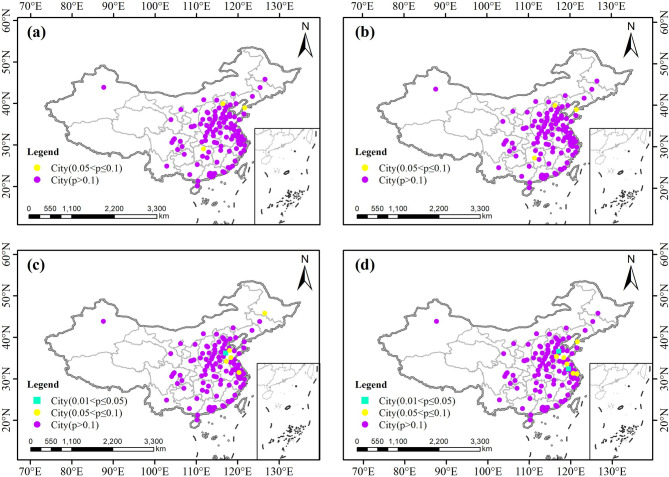
Significance levels of the causality test coefficients between monthly average air pollution and PEC. (**a**) PEC to PM_2.5;_ (**b**) PM_2.5_ to PEC; (**c**) PEC to AQI; (**d**) AQI to PEC.

As illustrated in Figs. 9 and 10, under weekly and monthly data scales, cities with significant causal links between air pollution and PEC remain predominantly located in the economically advanced North China Plain and the Yangtze River Economic Belt. This result further indicates that PEC is not a universally applicable feedback mechanism for pollution mitigation, and its effectiveness largely depends on regional economic development levels and governance capacity, mainly manifesting in economically developed areas. However, data at different temporal scales exhibit significant differences in characterizing the relationship between pollution and public environmental concern. First, daily average data can more accurately capture the short-term dynamic relationship between air pollution and public responses. By directly matching daily pollution levels with changes in public concern, information loss caused by temporal aggregation can be effectively avoided, thereby allowing clearer identification of the immediate effects of pollution exposure on public behavior. Moreover, daily data help mitigate identification bias caused by measurement errors and omitted variables, improving the accuracy and timeliness of model estimation. Existing studies show that public concern typically increases gradually after pollution events and declines after reaching a peak, with a lag of 0–4 days, further highlighting the importance of high-frequency data in capturing short-term response mechanisms. Although weekly or monthly averages have advantages in reflecting long-term trends (such as policy implementation or behavioral inertia), their aggregation process often involves averaging that smooths or even masks short-term fluctuations in the original data. Particularly under extreme pollution events or sudden environmental risks, key dynamic information may be significantly weakened, making it difficult to accurately reflect the temporal heterogeneity and nonlinear relationship between pollution exposure and public responses.

In summary, although causal relationships between pollution and public environmental concern differ somewhat across weekly and monthly scales, the empirical results are largely consistent with those based on daily data, indicating strong robustness across temporal scales.

## Discussion

**Fig. 11:**
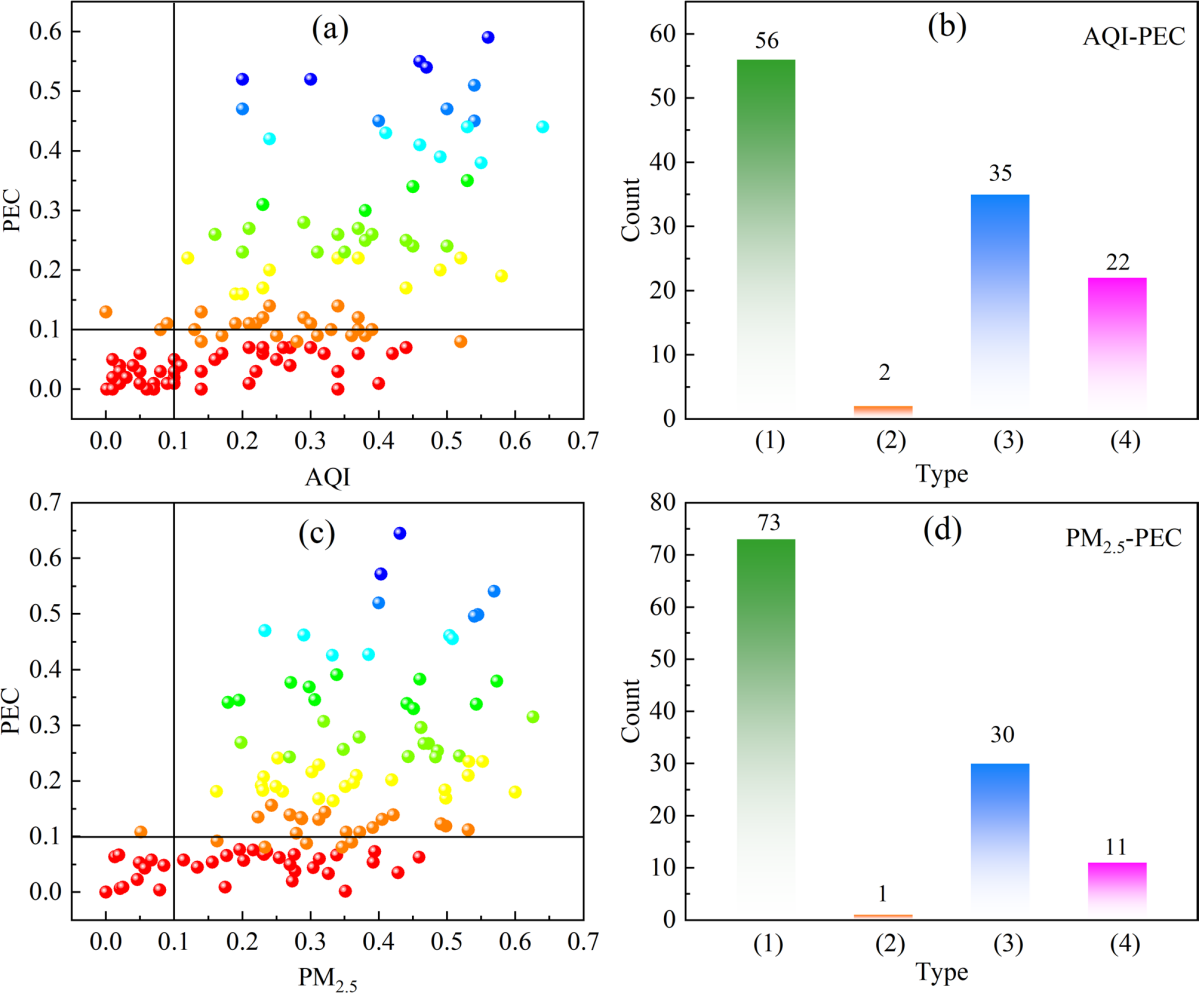
The combined causal relationship between air pollution and PEC.

From a city-level perspective, the causal relationship between PEC and air pollution can be classified into four categories. (1) If neither the CCM correlation from PEC to air pollution nor the reverse passes the significance test, no causal relationship exists between the two. (2) If the CCM correlation from PEC to air pollution is significant, but the reverse is not, then PEC is said to influence air pollution. (3) If the CCM correlation from air pollution to PEC is significant, but not vice versa, then air pollution influences PEC. (4) If both directions pass the significance test, then there is a bidirectional causal relationship between PEC and air pollution. Figure 11 presents the number of cities falling into each of the four causal categories based on CCM tests between PEC and AQI(PM_2.5_) under 10% significance level.

In practice, most urban residents only become concerned about air pollution when pollution levels exceed established standards. The AQI effectively indicates both the primary pollutant and the overall pollution intensity. To visualize the relationship between key pollutants and PEC, four causal interaction categories were calculated and illustrated in Fig. 11(a)-(b). Figure 11(a)-(b) show that 22 cities (19.13%), such as Beijing, Chengdu, Handan, Hangzhou, Hefei, Jinan, and others, exhibit bidirectional causality between AQI and PEC. Tai’an and Chongqing cities show a unidirectional causal relationship from PEC to AQI (1.74%). A unidirectional causal relationship from AQI to PEC was identified in 35 cities, representing 30.43% of the total sample, while no causal link was detected in the remaining 56 cities (48.70%). In cities where a bidirectional causal relationship exists between PEC and AQI, the two variables mutually exert mutual influence. In these cities, rising air pollution stimulates an increase in PEC, as heightened environmental awareness subsequently contributes to pollution mitigation. Thus, PEC can function as a key mechanism for air pollution control within broader environmental governance efforts. In cities where AQI cause PEC, PEC does not serve as a pathway for pollution governance. However, improving air quality can diminish PEC. For most cities, there is no causal relationship between air pollution and PEC. PM_2.5_ is one of the primary pollutants responsible for haze events. In daily life, direct physical sensory experiences play a key role in raising public awareness^10^. As air pollution worsens, a growing number of individuals become aware of air quality issues via direct experiences such as visual haze and noxious smells. Haze events that are directly perceivable tend to provoke greater public concern for environmental issues. Accordingly, the study further investigates the four causality combinations between PM_2.5_ and PEC. As shown in Fig. 11(c)-(d), a bidirectional causal link between PEC and PM_2.5_ is observed in 11 cities, accounting for only 9.57% of the total sample. Xi’an is the only city where a one-way causal relationship from PEC to PM_2.5_ is observed, comprising merely 0.87% of all cities examined. PM_2.5_ is found to unilaterally influence PEC in 30 cities, accounting for 26.09% of the sample. No causal relationship between PEC and PM_2.5_ is observed in the remaining 73 cities, which constitute 63.47% of the total sample. In cities where bidirectional causality exists between PEC and PM_2.5_ pollution, the two variables mutually influence one another. That is, increasing air pollution raises PEC, while heightened PEC helps suppress pollution. In these cities, PEC can be strategically utilized in environmental governance as a mechanism to manage PM_2.5_ pollution. For cities in which PEC cause PM_2.5_, increasing public attention to environmental issues may contribute to air pollution reduction. However, Xi’an is the only city exhibiting this causal direction. In cities where PM_2.5_ causally affects PEC, PEC cannot be utilized as a governance strategy. However, controlling PM_2.5_ pollution can reduce the intensity of PEC. Most cities do not exhibit a significant causal relationship between air pollution and PEC. In certain cities, the causality structures between AQI and PEC differ from those between PM_2.5_ and PEC. This is likely due to inconsistencies in the dominant air pollutants across cities. However, in most cities, the AQI-PEC causality combination subsumes that of PM_2.5_-PEC. There are two main reasons. First, PM_2.5_ remains the primary pollutant in China. Second, haze events are the primary environmental incidents that trigger PEC.

## Conclusion

Based on AQI and PM_2.5_ concentration data as well as public environmental concern (PEC) data from 115 prefecture-level cities in China between January 1, 2023 and December 31, 2024, this study employs the Convergent Cross Mapping (CCM) method to investigate the causal relationship between PEC and air pollution. Through empirical analysis, this study draws three main conclusions. First, PEC and air pollution exhibit significant nonlinearity and weak coupling characteristics in the short term. The correlation between PEC and air quality (PM_2.5_) shows an intermittent pattern with alternating positive and negative relationships. Second, CCM results indicate that in most cities, worsening air pollution stimulates public concern about environmental issues, while the contribution of increased PEC to improving air quality is minimal. Finally, cities where strong causal relationships between air pollution and PEC exist are mainly concentrated in the economically developed North China Plain and the Yangtze River Economic Belt. Based on the above findings, this study proposes the following policy recommendations. First, the nonlinear and weakly coupled relationship between PEC and air pollution indicates that the effectiveness of public supervision is unstable and its governance impact may be difficult to sustain. Meanwhile, the alternating positive and negative correlation reflects two distinct behavioral patterns of the public in environmental governance. In the negative correlation phase, where public concern is high and pollution levels are low, public attention effectively plays an external supervisory role. In contrast, during the positive correlation phase, both PEC and pollution levels increase simultaneously, reflecting public expression of environmental risk concerns or passive responses to sudden pollution events. Therefore, relevant authorities should establish efficient response systems to systematically collect, standardize, and professionally process public concern information. Given the significant short-term fluctuations in the relationship between PEC and air pollution, policy focus should shift toward building stable institutional frameworks to transform public supervision from temporary, event-driven participation into normalized and institutionalized mechanisms. Second, PEC should not be regarded as a universal feedback mechanism for mitigating air pollution, as its effectiveness appears limited to economically developed urban areas. The strong causal relationship between PEC and air pollution in developed regions confirmed in this study highlights the supplementary role of social media data in environmental monitoring, but due to regional differences, PEC as a bottom-up feedback mechanism must be applied with context-specific considerations. In developed regions, this supervisory effect should be strengthened, institutionalized, and standardized. In less developed regions, this model should not be imposed; instead, government responsiveness should be strengthened to lower participation barriers and reduce governance imbalances caused by uneven economic development.

In addition, future research can proceed in the following three directions. First, expand the sample coverage to compare heterogeneity in the causal relationship between PEC and air pollution across regions, city sizes, and industrial structures, and clarify the moderating effects of economic development and industrial composition. Second, integrate perspectives of social structure and environmental cognition to analyze PEC differences among various social groups (e.g., by age, education, and income) and their impact on pollution governance. Third, extend the observation period and construct long-term panel data to examine the long-term evolution of the causal relationship between PEC and air pollution, while analyzing the impacts of urbanization, industrial upgrading, environmental policies, and extreme weather.

## Electronic Supplementary Material

Below is the link to the electronic supplementary material.

Supplementary Material 1

## Data Availability

All data supporting the findings of this study are available within the paper and its Supplementary Information.
